# Malnutrition in Substance Use Disorders: A Critical Issue in Their Treatment and Recovery

**DOI:** 10.3390/healthcare13080868

**Published:** 2025-04-10

**Authors:** Joaquín García-Estrada, Sonia Luquin, Karen Pesqueda-Cendejas, Adolfo I. Ruiz-Ballesteros, Bertha Campos-López, Mónica R. Meza-Meza, Isela Parra-Rojas, Rocío Elizabeth González-Castañeda, Omar Ramos-Lopez, Ulises De la Cruz-Mosso

**Affiliations:** 1 Departamento de Neurociencias, Centro Universitario de Ciencias de la Salud, Universidad de Guadalajara, Guadalajara 44340, Mexico; jgarciaestrada@gmail.com (J.G.-E.); sonia.luquin@academicos.udg.mx (S.L.); karen.pesqueda20@gmail.com (K.P.-C.); monica.meza@academicos.udg.mx (M.R.M.-M.); rocio.gcastaneda@academicos.udg.mx (R.E.G.-C.); 2 Instituto de Neurociencias Traslacionales, Departamento de Neurociencias, Centro Universitario de Ciencias de la Salud, Universidad de Guadalajara, Guadalajara 44340, Mexico; berth.clampez@gmail.com; 3 Red de Inmunonutrición y Genómica Nutricional en las Enfermedades Autoinmunes, Centro Universitario de Ciencias de la Salud, Universidad de Guadalajara, Guadalajara 44340, Mexico; adolfo.ruba@gmail.com (A.I.R.-B.); iprojas@yahoo.com (I.P.-R.); 4 Laboratorio de Investigación en Obesidad y Diabetes, Facultad de Ciencias Químico-Biológicas, Universidad Autónoma de Guerrero, Chilpancingo de los Bravo 39087, Mexico; 5 Red Iberoamericana de Colaboración Académica y Científica en Nutriómicas y Nutrición de Precisión (RINN22), 28049 Madrid, Spain; 6 Facultad de Medicina y Psicología, Universidad Autónoma de Baja California, Tijuana 22390, Mexico

**Keywords:** malnutrition, substance use disorders, craving, drugs, nutrients, addictions, nutrition, eating behavior

## Abstract

Substance use disorders (SUDs) are widely prevalent in many countries, with the highest rates observed in nicotine and alcohol use, followed by opioid and cannabis use disorders. Within the field of SUDs, nutrition has become an increasingly important area of focus in both epidemiology and public health, as malnutrition is frequently observed among individuals affected by these disorders. Research indicates that people with SUDs are more likely to experience malnutrition than the general population; however, this issue remains an often-overlooked consequence that can impact disease progression and recovery outcomes. SUDs disrupt brain metabolism, leading to changes in brain function and disturbances in glucose, protein, and lipid metabolism. Evidence shows that individuals with certain SUDs often suffer from poor nutritional status, marked by high sugar consumption and insufficient intake of key micronutrients like iron, as well as vitamins D, C, A, and B—likely due to prioritizing drug use over adequate food intake. Importantly, diet can alter the metabolism and effects of drugs, potentially amplifying or diminishing their impact. While nutrition should play a central role in SUD treatment and rehabilitation, current research—both in animal models and human studies—on the role and benefits of specific nutrients in this context remains limited. This literature review aims to synthesize the available findings on the impact of malnutrition in human and murine models of SUDs, with the goal of identifying which nutrients may provide the most support for treatment and recovery.

## 1. Introduction

Substance use disorders (SUDs) are highly prevalent across most countries; the prevalence is highest for alcohol and nicotine, followed by opioid and cannabis use disorders. The impact of SUDs on society extends across multiple dimensions, including health and mortality, economic consequences, and crime [1]. Drugs contribute to many acute and chronic diseases, such as infectious, pulmonary, metabolic, cardiovascular, psychiatric, and oncological diseases [1].

Nutrition in the context of SUDs represents a critical and growing area of interest. It has been shown that drug abuse can affect eating patterns; in this sense, people with SUDs usually consume quick, cheap, and sweet foods, eat infrequently, and have little interest in food, contributing to the develop of malnutrition, characterized by essential micronutrient deficiency and sometimes by the development of obesity [2,3,4]. It has been also reported that nutritional deficiency in combination with drug abuse may increase the risk of developing metabolic syndrome by augmenting cell damage and excitotoxicity, reducing energy production, and lowering the antioxidant potential of cells [5]. Also, diet can influence how drugs are metabolized by increasing or decreasing their effects; therefore, inadequate nutrition could be a risk factor during drug abuse, provoking disease pathogenesis [5].

Malnutrition is a major yet under-recognized consequence of SUDs, influencing disease progression and recovery; malnutrition in SUDs includes undernutrition, overnutrition, and disease-related dysnutrition. Undernutrition is common, with a 24% to 50% prevalence of mild/moderate malnutrition in SUD patients; poor appetite and diet quality are prevalent, with 88% of patients requiring nutritional guidance [2]. Micronutrient deficiencies are widespread, with low levels of vitamin A (21%), iron (18%), potassium (12%), thiamine, folate, vitamin D, antioxidants, vitamin B12, and vitamin C (8%), which can lead to conditions such as alcoholic myopathy, osteopenia, osteoporosis, and mood disorders [2,6,7]. Low skeletal muscle mass and starvation-related malnutrition are prevalent in hospitalized patients [8,9]. Some individuals, particularly those in recovery or using appetite-stimulating substances, may experience overnutrition, which can lead to obesity [2].

Notably, evidence in humans and animal models demonstrated that healthy dietary patterns, such as the Mediterranean diet [10] and supplementation with vitamin D [11] as well as selenium, could improve some symptoms and complications in people with SUDs [12]. However, studies in this field are limited and, currently, there are no specialized training programs for the nutritional approach to substance use in addiction treatment settings [2,3,4].

Therefore, diet can influence how drugs are metabolized by increasing or decreasing their effects; although nutrition should be part of the treatment of and recovery from SUDs, current evidence in murine models and humans about the impact on and role of some nutrients in SUD treatment and recovery remains sparse. Hence, this literature review aims to synthesize the available findings on the impact of malnutrition in human and murine models of SUDs, with the goal of identifying which nutrients may provide the most support for treatment and recovery.

## 2. Materials and Methods

A compressive literature search was performed in selected databases and search engines—PubMed, Europe PubMed Central, and Scielo—considering the period from 1955 to 2023. The following keywords were used to obtain information on the topics and subtopics: “Substance use disorders”, “substance use disorders” AND “nutritional complications, “substance use disorders” AND “nutritional status” “malnutrition” AND “substance abuse”, “nutrition” AND “alcoholism”, “food addiction”, “cocaine” AND “malnutrition”, “methamphetamines” AND “malnutrition”, “opioids” AND “malnutrition” “substance use disorders treatment”, “substance use disorders” AND “diet”, “substance abuse” AND “nutritional deficiencies”. Likewise, the methodology and quality of the articles were carefully reviewed, and a complementary bibliography of each selected article was used to find more relevant information. Only English articles were considered. Errata, letters, comments, editorials, and duplicate articles were excluded. The selected articles for this literature review included original articles; observational and descriptive studies; systematic reviews; critical reviews; meta-analyses; and clinical trials in human and murine models.

## 3. Malnutrition in the Context of SUDs

The SUDs affect brain metabolism in specific regions by altering neurotransmitter signaling and influencing brain activity, causing alterations in glucose, protein, and fatty acid metabolism [5]. Several environmental factors including dietary habits affect the response to drug abuse; for example, chronic food restriction increases the locomotor stimulant effect of drugs, and nutritional alterations are reflected through metabolic changes that manifest as behavioral changes [5]. The relationship between eating behavior and the effects of cocaine and other psychoactive drugs can be attributed, in part, to the role of specific amino acids, such as phenylalanine, tyrosine, and tryptophan, which serve as precursors to key neurotransmitters such as dopamine, norepinephrine, and serotonin. These neurotransmitters regulate reward, motivation, and appetite, suggesting that the availability of amino acids in the diet may influence eating behavior and drug-related effects [13]. Some psychostimulants such as amphetamine, fenfluramine, and cocaine have been shown to reduce food intake in a nutrient-dependent manner. Human studies reveal that individuals with SUDs frequently experience calorie and protein malnutrition, which is associated with decreased enzyme cholinesterase activity (which metabolizes cocaine), increasing the risk of cocaine toxicity [13].

Therefore, diet quality may indirectly influence serum cocaine levels and related systemic effects. In particular, studies in rats indicate that fenfluramine suppresses fat intake more than carbohydrate intake, whereas cocaine causes the transient suppression of all macronutrients, followed by compensatory suppression. This suggests that high-carbohydrate and high-fat diets may amplify the anorexic effects of these drugs, whereas higher protein intake appears to prevent compensatory hyperphagia. This suggests that dietary composition influences withdrawal behaviors, as high-fat diets are associated with a depression-like state during cocaine withdrawal. In contrast, high-protein diets appear to have protective effects [13].

Notably, drug abuse can lead to malnutrition and disturbances in nutrient absorption, as well as increased or decreased appetite. More than 60% of drug addicts suffer from multiple malnutrition, based on signs of nutrient deficiency and biochemical values [14]. A study conducted on drug addicts in Oslo, Norway, described a high consumption of sugar and an insufficient intake of essential nutrients; also, in this study, patients struggling with multiple addictions showed greater deficiencies due to malnutrition, showing that up to 70% of addicts suffered from vitamin D deficiency and low levels of vitamin C, and 50% had iron or vitamin deficiencies (the most common being vitamins A, C, and E) [15] (Table 1).

The nutritional status of people with SUDs is generally under-reported in the literature [20]. SUDs can lead to malnutrition and metabolic disorders that compromise the nutritional status of individuals by altering their body composition and mental health [21]. Therefore, nutrition should be an important part of the treatment of SUDs, as recovery outcomes can be improved by nutritional therapy and balanced nutrient intake [20,22].

People with chronic SUDs are often malnourished [23]. The main SUD is alcohol, which inhibits the direct absorption of many nutrients [24] and chronic alcohol use can also severely affect the health of the entire digestive system. Chronic alcohol consumption has been linked to pathological alterations of the gastrointestinal tract, such as damage to the mucosa of the mouth, esophagus, and stomach, delayed gastric emptying, increased intestinal permeability and damage to barrier membranes, dysbiosis, and cancer risk [25]. This severely affects the digestion and absorption of essential nutrients. As a result, nutrient deficiencies are common in this population, which has a deficiency or inadequate intake of most nutrients along with altered dietary patterns [7,26,27]

Obesity is a public health problem that has experienced a significant increase in its prevalence in recent decades [28]. Regarding the groups most susceptible to SUDs, it has been described that in adolescents (12 to 19 years), the weight greater than healthy grew from 34.9% in 2012 (21.6% overweight and 13.3% obesity) to 38.4% in 2018 (23.8% overweight and 14.6% obesity). As in adults, the proportion of cases with a weight greater than healthy is higher in women (overweight 27%, obesity 14.1%, total 41.1%) than in men (overweight 20.7%, obesity 15.1%, total 35.8%) [28]. Santolaria et al. reported that 49.3% of male alcoholics were found to be either underweight or overweight/obese; also, malnutrition was related to the intensity of ethanol intake, the development of social or familial problems, the irregularity of feeding habits, and cirrhosis with ascites [3] (Table 1).

### Pathophysiology of Malnutrition in People with SUD

**Figure 1:**
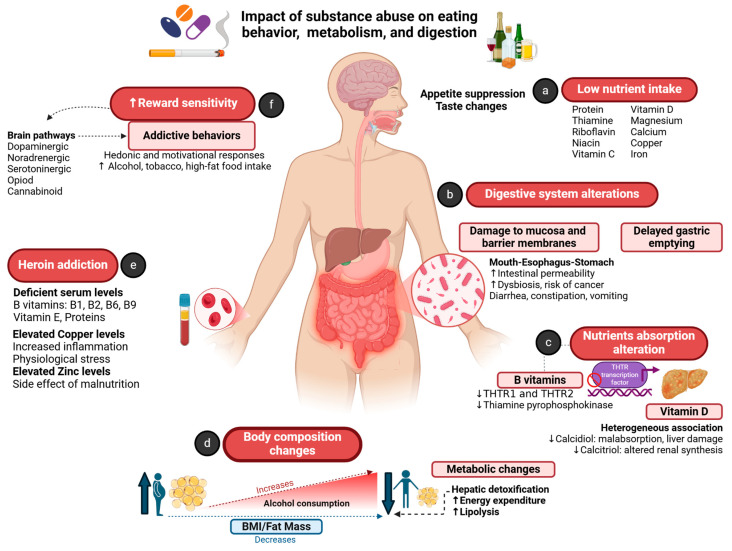
The impact of substance abuse on eating behavior, metabolism, and digestion. (**a**) Low nutrient intake (proteins, vitamins and minerals) influenced by appetite suppression and taste changes. (**b**) Digestive system alterations: damage to the digestive system mucus and barrier membranes (that causes an increase in the intestinal permeability, dysbiosis, risk of cancer), and delayed gastric emptying. (**c**) Alteration in absorption of nutrients such as thiamine (by reducing the transcription factors of THTR1 and THTR2, and limiting the production of thiamine pyrophosphokinase) and vitamin D (decreasing its absorption, hepatic calcidiol synthesis, and renal calcitriol synthesis). (**d**) Body composition changes: in the early stages of alcoholism, it increases BMI and fat mass. Later, chronic alcohol consumption is inversely correlated with BMI and fat mass (greater elimination of toxic substances by the liver leads to increased thermogenesis and reduces lipogenesis). (**e**) Heroin addiction: subjects with this condition present deficient levels of protein and vitamins, and elevated zinc and copper levels (due to inflammation, stress, and malnutrition). (**f**) Reward sensitivity is associated with substance abuse and a high-fat diet; several brain pathways involved in pleasure, reward, and motivation can influence addictive behaviors. Arrows ↑ and ↓ indicate increase and decrease, respectively. THTR: thiamine-absorbing transporters; BMI: body mass index. Created in biorender.com.

It has been described that people with high levels of alcohol consumption have a lower body mass index (BMI) and fat mass [29]. In the early stages of alcoholism, energy from alcohol increases BMI and fat mass; later, as alcohol consumption increases and becomes chronic, alcohol consumption is inversely correlated with BMI and fat mass [30,31]. However, the point at which this metabolic shift from the effect of alcohol occurs is unknown and may vary between individuals. Even a normal BMI among subjects with SUDs does not mean that they are healthy. There are several possible explanations for the paradox of increased energy intake in subjects with alcoholism and a lower-than-expected BMI and fat mass. Among the proposed hypotheses, it has been described that a greater elimination of toxic substances by the liver in alcoholics leads to greater energy expenditure [29]. Under specific conditions only—for example, fasting—preferred energy sources may shift from glucose to acetate in the brain, and there is even evidence of greater use of lipids as an energy source [32]. While a lower percentage of fat mass could be the result of reduced lipogenesis during alcohol intoxication, there is also some evidence of increased thermogenesis with higher alcohol intake [33]. Likewise, subjects with alcoholism often have nutritional deficiencies because their intake of protein, thiamine, riboflavin, niacin, vitamin C, vitamin D, magnesium, calcium, copper, and iron is below the reference nutritional values [27,34] (Figure 1).

The results of malnutrition assessment using anthropometric measurements are sometimes not very reliable. Thus, in addition to body assessment, measuring plasma levels of different nutrients helps to identify nutritional deficiencies associated with malnutrition. In injectable drug users, deficiencies in different essential nutrients have been identified, such as selenium and potassium (due to lower muscle mass), iron, and vitamins A, C, D, and E [20]. This malnutritional pattern is maintained in others subjects with SUDs; for example, in heroin-dependent subjects, levels of protein, folate, thiamine, riboflavin, B6, and vitamin E have been described as deficient [35]. Elevated levels of some minerals have also been reported, such as phosphorus, sodium, and magnesium (possibly due to partial dehydration), as well as copper and zinc (due to acute fasting and smoking) [20]. The elevated copper levels described in subjects with SUDs [15,34] may be due to increased inflammation and the presence of physiological stress, since serum zinc levels may be higher than in the general population, suggesting that this may be a side effect of malnutrition (Figure 1).

The reasons for the presence of malnutrition in these subjects may be due to the choice of drugs over food consumption [21,36]. However, other pathophysiological and food insecurity factors, such as appetite suppression, taste changes, and a lack of money, motivation, or cooking facilities, have also been observed to influence the development of malnutrition [36]. Unlike alcohol, drugs do not specifically compromise the structure of the gastrointestinal tract; however, users experience digestion and absorption difficulties, often through diarrhea, constipation, or vomiting [36]. In addition, opioids reduce motility in the gastrointestinal tract; therefore, malnutrition is also common among this population [27] (Figure 1).

## 4. Eating Behavior: Overlap of Food Addiction and SUDs

Notably, there is a relationship between the psychobiological processes of SUDs and eating behavior: both processes share brain circuits that mediate reward, salience, and motivation [37]. Both are influenced by emotional states and stress [27]. Furthermore, an overlap in personality traits, and other common liabilities associated with risk for SUDs and food addiction, has been reported [38]; for example, reward sensitivity is associated with increased alcohol and tobacco use and high-fat food intake [39]. A defining feature of drugs of abuse is that they strongly activate dopaminergic, noradrenergic, serotonergic, opioid, and cannabinoid brain pathways involved in pleasure, reward, and motivation [40]. These same pathways are linked to hedonic and motivational responses to eating [41].

Energy is the primary reinforcing quality of food, and the brain is extremely sensitive to its supply [41]. Increasingly, animal models of binge eating on sweet and palatable foods display addictive behavior, which is mediated by dopamine and endogenous opioid neurotransmitters in these brain circuits [27] and requires both stress and hunger to be present [42]. Both the overconsumption of energy-dense foods in animal models and palatability and intake of foods in humans are reduced by nonspecific opioid antagonists, such as naloxone and naltrexone, and selective opioid antagonists [27]. In contrast, opioid agonists can potentiate the binge eating of palatable foods in stressed, food-restricted rat models [27,42]. There is also evidence to suggest that drug withdrawal-like behavior is manifested when access to sweet, palatable foods is restricted [27], and that “binge eating” on high-fat foods can alleviate opioid withdrawal in rats and enhance alcohol motivation in mice [27]. Furthermore, it has been argued that some foods, particularly those with a high sugar content, may have “addictive potential” for some individuals [43]. (Figure 1).

It has been demonstrated that the excessive consumption of glucose or fructose promotes neurological changes in areas of the brain associated with reward, learning reward, and eating behavior. In this sense, it was observed that glucose had a large impact on the brain by affecting mood and promoting compulsivity and impulsivity repetitive behaviors, which are SUD predictors [44]. Therefore, high consumption of sugar can be addictive and can predispose to the risk of food addiction.

Foods are highly rewarding stimuli for individuals; evidence suggests that the consumption of sugar generates the activation of the mesocorticolimbic system reward and other systems involved in the regulation of intake [45]. According to Winterdahl et al., the excessive consumption of sugars decreases the availability of opioid receptors and dopaminergic receptors (D2 and D3) in structures of the mesocorticolimbic reward system, such as the nucleus accumbens, thalamus, amygdala, cingulate cortex, and the prefrontal cortex [46]. However, addiction to food or sugars is still a controversial diagnosis that is not included in current systems qualifiers created by the American Association of Psychiatry or the World Health Organization [45].

### Hunger and Craving Related to SUDs

Drug addicts who are malnourished experience cravings but may find it difficult to differentiate between urges to consume addictive substances and those driven by the need to eat, a phenomenon called “addiction transfer” [47]. Part of this phenomenon could be because craving or seeking relief from a need is survival behavior under the influence of reward pathways in the brain, which in turn are altered by nutritional need [47]. Therefore, food deprivation lowers the threshold for the activation of reward pathways, which increases sensitivity to drugs of abuse as well as food [27,37], potentially further reinforcing the consumption of either. In contrast, cocaine withdrawal devalues the sweet taste, mediated by accumulated dopamine [27]. In this way, nutrient deficiencies may also contribute to cravings or at least encourage drug seeking, as nutrient-deprived animals seek out new reinforcing experiences, mediated by brain dopamine activation [27]. Appetite expressions, such as hunger and cravings, are regulated by anorexigenic and orexigenic peptide hormones. Given the already-described overlap between drug, alcohol, and food appetite, it can be expected that SUDs may affect and be affected by these hormones [27]. These interactions are now considered for the peripherally released, but centrally active, hormones ghrelin, leptin, and insulin. Although many other neurohormones could be involved, data on human addicts are scarce [48].

## 5. Nutritional Approaches to the Treatment and Recovery of SUDs

The first nutritional approach to drug use was proposed in 1955 [49]; however, it was not until 1990 that the American Dietetic Association (now the Academy of Nutrition and Dietetics) specifically proposed that “nutritional interventions are an essential component of the treatment and recovery of SUD” [50]. It should be noted that these recommendations have not been implemented by the health sector and the participation of nutrition professionals in treatment settings for SUDs has not been encouraged. It is a fact that people with severe SUDs may have inadequate dietary intake, which can lead to malnutrition [15,51]. Despite there being no specialized training programs addressing the nutritional approach to substance use in addiction treatment settings, in recent years, private addiction treatment centers have begun to incorporate “holistic” approaches that pay special attention to healthy eating and emphasize the importance of nutrition during substance use recovery and treatment. However, there are no established standards of practice [52]. This could be because nutritional treatment for SUDs is conditioned by the type of substance used, as well as the severity and period of substance use.

### 5.1. Alcohol and Alcoholism as a Trigger for Malnutrition

Among the main SUDs, alcohol stands out as the most common initial drug; globally, alcohol use disorder affects over 100 million people, while opioid and cannabis dependence impact 26.8 million and 22.1 million individuals, respectively [53]. Alcoholism is one of the costliest health problems due to the health complications associated with excessive alcohol consumption and malnutrition is a common disorder in people with alcoholism [54]. The classic phenotype of malnutrition in people with alcoholism is characterized by the presence of sarcopenia [54]. These people have poor protein intake, altered fat intake, and a deficiency of some essential micronutrients with antioxidant, anti-inflammatory, and antifibrogenic functions. Patients with alcoholism usually consume an average of 10 to 15 drinks a day; thus, this intake becomes an important source of calories with little nutritional value [54]. The degree of malnutrition correlates positively with the classic complications of alcoholism such as encephalopathy, ascites, hepatorenal syndrome, and risk of mortality [54]. It has been reported that approximately 84% of patients with alcohol-associated cirrhosis have malnutrition [54]. These data demonstrate the need for nutritional treatment in the approach to patients with alcoholism [54]. In patients who underwent treatment for alcohol and drug abuse, the prevalence of mild/moderate malnutrition was found to be 24% [2] (Table 1).

The main components for a targeted intervention to prevent sarcopenia and maintain adequate muscle mass are diet quality, protein intake, adequate hydration, and the supply of nutrients with anti-inflammatory properties [2]. The Mediterranean diet is associated with greater muscle strength and function due to its balanced content of vitamins and antioxidants [2].

It is essential to ensure adequate protein intake within the recommended intake amounts: for prevention in healthy adults, this amount ranges from 0.8 to 0.9 g protein/kg of body weight, but for individuals with alcohol abuse and sarcopenia, protein intake should not be less than 30–50 g per day; this is similar to the recommended intake of 1.0 to 1.2 g/kg/day for older adults (>65 years of age) [55,56,57]. In addition to the amount of protein, it is important to consider the quality of the protein, expressed in the digestible indispensable amino acid score (DIAAS), which is based on the ileal digestibility of protein [55]. Most animal food sources provide protein of excellent quality (DIAAS ≥ 100) compared to soy, whey (high quality, DIAAS = 75–99), and other plant sources (DIAAS < 75), although combinations of different plant proteins can achieve excellent quality scores [55].

Moreover, chronic alcohol consumption can promote gastrointestinal problems, affecting digestion and the absorption of essential nutrients such as thiamine, riboflavin, niacin, pyridoxine, folic acid, vitamins A, C, D, E, and K, magnesium, and selenium. Between 30 and 80% of alcoholics have a thiamine deficiency, which increases their risk of developing Wernicke–Korsakoff syndrome [58,59]. Furthermore, if alcohol intake exceeds 30% of total caloric intake, it is common for carbohydrate, protein, and fat intake to be significantly reduced; in addition, vitamin consumption (A, C, and B1 or thiamine) is also below the minimum dietary reference intake limits [57]. The development of osteoporosis is another complication related to excessive alcohol consumption. Approximately 34% to 48% of people with alcoholism have osteopenia. Vitamin D is necessary for calcium absorption and has an important role in immune function. Thus, these patients should be monitored to ensure adequate consumption as well as serum levels of this vitamin [60,61,62].

Therefore, alcohol is one of the main drugs of abuse that inhibits thiamine absorption, reducing the transcription factors of thiamine-absorbing transporters in brush border cells [63]. Furthermore, alcohol limits the production of thiamine pyrophosphokinase, an enzyme that converts thiamine into thiamine pyrophosphate, which is a coenzyme for metabolic functions. Thiamine deficiency has been associated with cognitive dysfunction [63]. In addition to B vitamins, levels of other vitamins are also affected by alcohol intake, such as vitamin D, for which a high prevalence of hypovitaminosis D has been described in these subjects [64,65]. However, evidence of the association between alcohol dependence and vitamin D is heterogeneous, as both positive and negative correlations have been described [66]. The presence of low calcidiol levels (25-OH D) may be the result of low sun exposure and food intake, decreased levels of the vitamin D-binding protein (VDBP), malabsorption, altered biliary excretion, and alcohol-induced liver damage (reduced hydroxylation capacity). In another scenario, calcidiol levels may be elevated and there may be low calcitriol (1,25-dihydroxyvitamin D, active metabolite of vitamin D) levels; it has been proposed to explain this that excessive alcohol consumption may block the conversion of calcidiol into calcitriol (altered renal synthesis) and/or increase calcitriol degradation [67,68,69].

Patients with chronic alcoholism often have folate deficiency due to inadequate dietary intake, poor intestinal absorption, increased urinary excretion, and reduced hepatic storage. Folate deficiency favors the progression of liver disease through alterations in methionine metabolism, which alters DNA synthesis and stability and affects the epigenetic regulation involved in liver injury pathways [70]. Zinc deficiency is common in alcohol-related liver disease and contributes to Paneth cell dysfunction, disrupting antimicrobial defense and promoting bacterial translocation [71]. This exacerbates gut dysbiosis, systemic inflammation, and liver damage, highlighting zinc’s crucial role in maintaining intestinal and hepatic homeostasis in substance use disorders [71].

Therefore, zinc supplementation plays a crucial role in addressing alcohol-associated hepatitis. Due to the presence of vomiting, diarrhea, excessive urinary loss, and an inadequate diet, hypomagnesemia is another frequent complication in alcoholics, which can manifest in muscle cramps and can be reversed with supplementation [72].

Regarding nutritional care concerning the effects of excessive alcohol consumption, a meta-analysis carried out in 2014, which included 13 clinical trials in which nutritional therapies were carried out on patients with alcoholic or cirrhotic hepatitis, showed that in those patients who received nutritional therapy, mortality was reduced and hepatic damage, encephalopathy, and the presence of infections were prevented [73]. There is also evidence that probiotics, prebiotics, and even fecal matter transplants are recommended to repopulate the intestinal microbiota in the presence of alcoholic liver disease [74]. In murine studies, it has been observed that olive oil polyphenols exert a protective effect on alcohol-induced oxidative stress [75]. It is noteworthy that patients who present chronic excessive alcohol consumption show a high preference for sweet foods and sugary drinks, possibly explained by the relationship between carbohydrate consumption and serotonergic transmission [52]. Moreover, the association between nutrients and neural plasticity has been previously described; specifically, alterations in animal models’ fat consumption through a ketogenic diet decrease ethanol consumption by changing gene expression in dopamine, adenosine, and cannabinoid systems [76]. Therefore, nutritional interventions may be a useful complementary tool in subjects with alcoholism (Table 2).

### 5.2. Cocaine

In a 2010 Canadian study of people who were intravenous drug users, 65% of these people met the criteria for hunger, which was strongly correlated with depression [82]. Likewise, in a study that evaluated 140 patients addicted to cocaine or heroin, 18% of them presented severe malnutrition (serum albumin less than 3.5 g/dL and/or serum transferrin less than 200 mg/dL) and this phenomenon occurred more frequently in women compared to men [83]. Also, in a study carried out on women addicted to heroin diagnosed with HIV, malnutrition was observed in all patients when they used this drug; however, improvement in their nutritional status was observed after 6 months of a “therapy detoxification”, during which they did not consume a variety of drugs [84].

Cocaine use is associated with alterations in the physiology of the intestine, brain, and endocrine system [85,86,87]. While it is not clear whether some of the observable alterations precede drug use, nutritional therapy designed to restore intestinal microbiota, stabilize hormones, and reduce addictive neurological circuits seems promising in the treatment of patients who present cocaine abuse. In a study in cocaine-dependent patients, N-acetylcysteine (NAC) supplementation decreased cue-induced craving in cocaine-dependent individuals [80] (Table 2). The effects of NAC in cocaine-dependent patients are attributed to its antioxidant activities. It is well known that the effects of oxidative stress in the nervous system have evolved from strictly neurotoxic to include a more nuanced role in redox-sensitive signaling. Specifically, S-glutathionylation, a redox-sensitive post-translational modification, has been suggested to influence the response to drugs of abuse. NAC stimulates cysteine–glutamate exchange and may restore glutathione (antioxidant), which appears to be related to addiction-signaling proteins and may have treatment benefits in cocaine addiction and other SUDs [88] (Table 2).

### 5.3. Opioids

Like cocaine, opioids are associated with hormonal abnormalities, including decreased serum leptin and rapid weight gain during treatment [89]. Attempts to alleviate withdrawal and mood symptoms during recovery using supplements containing neurotransmitter precursors (tyrosine, lecithin, L-glutamine, and 5-HTP) have shown promising results [78]. In a study of patients on methadone maintenance treatment (MMT), those receiving 50,000 IU of vitamin D every 2 weeks for 12 weeks showed significant improvements in sleep quality and decreased depression [11]. However, nutrient deficiencies should be confirmed by laboratory testing before excessive supplementation [52] (Table 2).

### 5.4. Methamphetamines

Methamphetamine use is commonly associated with decreased BMI and reduced production of neuropeptide Y (orexigenic hormone that promotes appetite), and it has even been associated with bulimia nervosa [90,91,92]. There is a link between methamphetamine use and oral health. Effects on dental health have the potential to impact all areas of nutrition by influencing food choices.

Likewise, increased oxidative stress was observed in polysubstance users, where methamphetamine-induced neurotoxicity was reduced with the use of selenium as an antioxidant [12]. The use of antioxidants in the form of supplements seems to be a promising approach to methamphetamine abuse because there are challenges in implementing a nutritional therapy focused on whole-plant foods with high fiber content and high antioxidant potential. Like alcohol and other intoxicating drugs, high-dose methamphetamine use disrupts epithelial barrier function by modulating tight junction integrity and epithelial cell viability [93]. Therefore, nutritional therapies aimed at restoring these gastrointestinal disorders are needed, such as the administration of prebiotics and probiotics (Table 2).

## 6. Discussion

Regarding treatments and strategies to improve the nutritional status of people with SUDs, workshops and educational interventions have been carried out worldwide to improve the quality of life of these people concerning nutrition. In a program carried out in the United States with prisoners with SUDs, using nutrition workshops, better nutrition practices and up to 4 times improvement in general health were observed after this intervention [94]. Another study in Italy showed that group nutritional education in a residential alcohol treatment center was associated with better nutritional behaviors (eating more than 3 meals a day) and a self-reported abstinence rate of 80% after 6 months [95]. Additionally, a 6-week environmental and educational intervention (including cooking activities) to improve dietary intake at 6 residential drug treatment centers for men in upstate New York found improved body composition, eating behavior, and overall satisfaction with education following this intervention [96].

Voracious food consumption may be due to a “rebound appetite” following hypothalamic suppression caused by SUDs. Making healthy food choices after achieving abstinence can be very difficult. Abstinence is associated with new emotions, anxiety, and uncertainty. It is easy to seek a predictable, comforting response in food. This can lead to overeating, relapses, compromised quality of life, and the development of chronic diseases. Weight gain during SUD recovery should be monitored and controlled (gradual rather than drastic) to counteract associated adaptations in nutrition-related hormones [27]. Various authors agree that the desire to eat very tasty foods would be considered a form of addiction related to dopamine and opioids, but there is no consensus on the extent to which eating habits should be intervened on during the recovery of patients with substance use abuse [52]. This phenomenon is specifically observed in the literature of the Alcoholics Anonymous Association, established in 1939, where it suggests the use of chocolates and sweet foods in the early abstinence stage; however, this presents a paradox since recent evidence shows that patients in recovery have an interest in reestablishing a healthy diet, and it should be noted that a key point is that suggesting an “extreme” dietary plan or restrictive diet should not be promoted [52]. The restoration of nutritional status in the recovery of the patient with SUDs must go beyond the correction of vitamin/mineral status and body weight and must also consider the recovery of intestinal function and dysfunctional neurohormonal circuits. Future research should target the “gut–brain” axis in addiction by considering the vagus nerve, the production of neurotransmitters serotonin and dopamine in the gut, and the hypothalamic–pituitary–adrenal (HPA) axis, which may lead to new “psychobiotic” treatments designed to promote the proper functioning of these systems in patients. Finally, it should be noted that the abuse of intoxicating substances is associated with suboptimal eating patterns and nutrient deficiencies [52]. While food restriction may trigger relapses, an excess may perpetuate the cycle of addictive behavior. The best strategy seems to be to strike a middle ground between these extremes, which will require clinical expertise in nutritional treatment settings. To achieve this, exposure to highly palatable foods with addictive potential should be controlled and the consumption of high-fiber foods, for example, should be encouraged. The focus should be on what to eat, rather than what not to eat. Nutritional interventions should never be viewed as punishment, but rather as a useful component of the physiological healing process in these patients.

Considering the prominent role played by psychological factors in the recovery from substance abuse, nutritional interventions would be best implemented in conjunction with expert therapeutic approaches to support patients’ mental health and well-being and facilitate the transition towards healthy eating patterns during treatment and recovery. Dietician-led sessions to address nutritional concerns and subjective experiences in the recovery phase should be an integral part of the recovery process, along with adequate educational support to increase nutrition literacy in these patients and ensure the long-term efficacy of the recommended treatment options. The limitations of the present review, above and beyond those mentioned, such as the limited availability of human studies, are the sample sizes of the studies included, the heterogeneity of study populations, and the choice of different biomarkers of malnutrition such as albumin, which make it difficult to compare and translate results across studies and populations. Additional limitations include the accuracy and reliability of the methodological assessments chosen to evaluate malnutrition.

## 7. Conclusions

SUDs affect the brain metabolism of specific regions by altering neurotransmitter signaling and influencing brain activity. These changes can cause alterations in the metabolism of glucose, proteins, and fatty acids. The main nutritional complications associated with addiction are altered eating behaviors, characterized by changes in appetite, and increased malnutrition risk status depending on the type of SUD. Likewise, metabolism can present variations and be related to changes in weight, and digestive, gastrointestinal, or liver problems. Moreover, SUDs may promote deficiencies in some nutrients, contributing to many acute and chronic diseases such as infectious, pulmonary, metabolic, cardiovascular, psychiatric, and oncological disease risk.

Nutritional therapies in SUDs should include those focused on providing adequate amounts of macro- and micronutrients to address related deficiencies, the use of probiotics and prebiotics to reverse gastrointestinal disorders, the promotion of high-protein and -fiber foods to replace energy-dense diets, and educational support to encourage healthy dietary habits. Regarding this, nutrition is essential to improving recovery outcomes for people with SUDs, as it addresses both physical and mental health needs. Many of these individuals suffer from malnutrition due to poor dietary intake and impaired nutrient absorption, leading to deficiencies in crucial vitamins and minerals. Restoring these nutrients is vital to health and recovery. Good nutrition also directly impacts mental health, as nutrients such as folate and omega-3 fatty acids play a key role in brain function and emotional stability, helping to reduce anxiety and depression. Additionally, proper nutrition can reduce physical problems, such as liver damage, commonly associated with SUDs. Therefore, by adopting healthy eating habits and correcting nutritional deficiencies, individuals can improve resilience, reduce the risk of relapse, and enhance long-term recovery. Overall, integrating nutritional therapy into treatment offers a comprehensive approach to address the interconnectedness of physical health, mental health, and substance use recovery, leading to better outcomes.

Finally, knowledge of the importance of nutritional support in the recovery and treatment of SUDs opens an important area of study and intervention to support the recovery of SUD users.

## Figures and Tables

**Table 1:** Evidence of malnutrition in subjects with SUDs.

Author	Population	Substance	Results	Conclusion
Saeland et al., 2011 [15]	123 male and 72 female drug addicts from Norway.	Multi-drug users	Added sugar accounted for 30% of the energy intake. Sugar and sugar-sweetened food items were preferred by 61% of the respondents.	Drug addicts have a high risk of inadequacy of food and nutrient intake.
Ross et al., 2012 [2]	67 patients (48 male, 19 female) drug addicts from Australia.	Multi-drug users	The prevalence of mild/moderate malnutrition was found to be 24%. 50% of all subjects were deficient in iron or vitamins (low vitamin A levels in 21%, low iron levels in 18%, low-range potassium in 12%, and low vitamin C levels in 8%).	Chronic substance abuse affects the nutritional status and is associated with nutrient deficiencies and malnutrition.
Nazrul Islam et al., 2002 [14]	253 male drug addicts from Bangladesh.	Multi-drug users	The drug addicts had significantly lowered BMI *, hemoglobin, and serum total protein and albumin levels. 60% of drug addicts were suffering from multiple malnutrition.	Drug addicts have poor nutritional status. Multiple malnutrition or nutrient deficiency is prevalent among them.
Santolaria et al., 2000 [3]	181 hospitalized male alcoholics from Spain.	Alcohol	BMI was under 18.5 kg/m^2^ in 8.9%. Malnutrition was related to the intensity of ethanol intake, development of social or familial problems, irregularity of feeding habits, and cirrhosis with ascites.	Malnutrition related to alcoholism seems multifactorial in its pathogenesis.
Yazici et al., 2019 [16]	189 schizophrenia patients, 119 substance use disorder patients and, and 109 controls from Turkey.	Multi-drug users	The prevalence of vitamin B12 deficiency in the SUD group was significantly higher than that in the control group (28.3% vs. 11.5%). Compared with the control group, vitamin D and B12 levels were significantly lower in the schizophrenia group, and folic acid and B12 levels were significantly lower in the SUD group.	Several vitamin deficiencies appear to be common in both schizophrenia and substance use disorder patients.
Bemanian et al., 2022 [17]	666 participants drawn from outpatient opioid agonist therapy from Norway.	Opiods	57% of all subjects had vitamin D deficiency (<50 nmol/L), and 19% were severely deficient (<25 nmol/L).	Patients with severe substance use disorders have a high prevalence of vitamin D deficiency.
Madebo et al., 2022 [18]	672 SUD patients from Norway.	Multi-drug users	22% of the population had suboptimal B12 levels (<300 pmol/L) and 1.2% were deficient (<175 pmol/L).	People with SUDs have B12 suboptimal levels that might or might not be adequate for metabolic needs.
Bemanian et al., 2022 [19]	663 participants drawn from outpatient opioid agonist therapy from Norway.	Opioids	48% of the population had low serum folate levels (<10 nmol/L), and 23% were deficient (<6.8 nmol/L).	Injecting substances is associated with a reduction in serum folate over time.

* BMI: body mass index; SUD: substance use disorder.

**Table 2:** Substance use disorders and evidence of nutritional approaches.

Author	Substance	Study Design	Population	Nutritional Approach	Outcome
Evidence in humans					
Kirpich et al., 2009. [77]	Alcohol	Randomized, prospective clinical trial	66 adult alcoholics from Russia	Probiotic therapy of 0.9 × 10^8^ colony-forming unit (CFU) *Bifidobacterium bifidum* and 0.9 × 10^9^ CFU *Lactobacillus plantarum 8PA3* for 6 days.	Repopulation of the intestinal microbiota.
Lee et al., 2024 [10]		Retrospective cohort study	5725 alcoholic subjects from Korea	High adherence to the Mediterranean diet.	Decrease in the risk of steatotic liver disease, metabolic dysfunction-associated steatotic liver disease and alcohol-related liver diseases.
Ghaderi et al., 2017. [11]	Opioids	Randomized, prospective clinical trial	68 patients with methadone treatment from Iran.	50,000 UI of vitamin D every 2 weeks for 12 weeks.	Improvements in sleep quality and decreased depression.
Chen et al., 2012. [78]		Randomized, prospective clinical trial	83 detoxified heroin addicts from China.	50 mg/kg/day of supplements containing neurotransmitter precursors (tyrosine, lecithin, L-glutamine, and 5-hydroxytryptophan).	Reduction in withdrawal and mood symptoms during recovery.
Ardekani et al., 2018 [79]	Cigarette	Randomized, prospective clinical trial	54 heavy-smoker males from Iran.	5 capsules of fish-oil-derived omega-3 fatty acid supplements (containing 180 mg of eicosapentaenoic acid and 120 mg of docosahexaenoic acid) for 3 months.	Reduction in cigarette craving and oxidative stress index in heavy-smoker males.
LaRowe et al., 2007. [80]	Cocaine	Randomized, prospective clinical trial	Six subjects with criteria for cocaine dependence from EE.UU.	600 mg of N-acetylcysteine for 3 days.	Decreased cue-induced craving in cocaine-dependent individuals.
Evidence in murine models					
Carito et al., 2017. [75]	Alcohol	Experimental	Murine model	20 mg/kg of olive polyphenols for two months.	Protective effect on alcohol-induced oxidative stress.
Imam and Ali, 2000 [12]	Methampheta-mines	Experimental	Murine model	Supplementation with 0.5 mg/kg of selenium for one week.	Reduction in neurotoxicity.
Hakimian et al., 2019 [81]	Opioids	Experimental	Murine model	Omega-3 fatty acids-enriched diet.	Decrease in anxiety-induced opioid-seeking behavior.
